# Recent developments in the use of biomarkers as diagnostic tools in patients with systemic lupus erythematosus: a narrative review

**DOI:** 10.1016/j.ero.2025.11.025

**Published:** 2026-02-09

**Authors:** Tammo Brunekreef, Henny Otten, Jacob M. van Laar, Maarten Limper

**Affiliations:** 1 Department of Rheumatology & Clinical Immunology, UMC Utrecht, Utrecht, Netherlands; 2 Center of Translational Immunology, UMC Utrecht, Utrecht, Netherlands

## Abstract

Systemic lupus erythematosus (SLE) is a heterogeneous systemic autoimmune disease with a complex a multifactorial pathophysiology. Due to difficulty recognising patients with SLE in an early stage, diagnostic delay is very common, which can result in the accumulation of organ damage. Some immunological signs, such as the presence of autoantibodies, have previously been shown to be present in patients with SLE, even before the onset of the first symptoms. This makes these immunological signs a prime target to improve the diagnostic process. In this article, we review recent studies investigating the use of biomarkers for diagnostics, including autoantibodies, other serum biomarkers, biomarkers in other bodily materials, circulating RNAs, and gene expression profiles. We discuss their place in the pathophysiology of SLE and their potential to improve and expedite the diagnostic process for patients with SLE.

## INTRODUCTION

Systemic lupus erythematosus (SLE) is a systemic autoimmune disease characterised by a wide range of symptoms and immunological manifestations [1]. Because of the heterogeneity of the disease, SLE can be difficult to diagnose, often resulting in a significant diagnostic and treatment delay [2]. This can result in the accumulation of organ damage, increasing the burden of the disease for patients [3].

There is an unmet need for additional tools to improve and expedite the diagnostic process to diminish this delay. In the absence of diagnostic criteria for SLE, the European Alliance of Associations for Rheumatology (EULAR)/American College of Rheumatology (ACR) classification criteria, developed primarily for research purposes, are commonly used in clinical practice as a substitute to aid clinical decision making, blurring the lines of what would be considered a diagnosis of SLE in clinical practice [1]. The prediagnostic phase of SLE (‘pre-SLE’) is characterised by the absence or limited presence of SLE-related clinical symptoms, which are insufficient to establish a clinical diagnosis of SLE. These symptoms are often nonspecific and can be caused by a wide range of different causes. However, in patients with pre-SLE, some signs of autoimmunity, such as the presence of autoantibodies and interferons, have been shown to be present frequently during this preclassification phase. We show an overview of these signs of autoimmunity in patients with pre-SLE in the Table [[4], [5], [6], [7], [8], [9], [10], [11], [12], [13], [14], [15], [16], [17], [18], [19]].

**Table:** Signs of autoimmunity in patients with pre-SLE

Immune factor	Marker	References
*Autoantibodies*	ANA	[5,6,9,12]
	Anti-Ro	[5,6,7,8,9]
	Anti-La	[5,6,8,9]
	APl	[5,16,19]
	Anti-dsDNA	[5,6,8,9,12,14,17]
	Anti-Sm	[5,6,8,9]
	Anti-RNP	[5,6,8,9]
	Anti-chromatin	[8,9]
	Rheumatoid factor	[13]
	Anti-PCNA	[16]
	Anti-C1q	[117]
	Increased IgG:IgM ratio	[16,17]
*Interferon*	IFN-α	[8]
	IFN-γ	[9,18]
	IFN-Score-B	[11]
	IFN-Score-A	[11]
*Soluble markers*	IL-6	[9]
	IL-12p70	[9]
	IP-10	[9,10]
	IL-5	[9]
	IL-10	[19]
	TGF-β	[9,18]
	Complement levels	[14]
	BLyS	[18]
	SCF	[18]

ANA, antinuclear antibodies; APl, antiphospholipid antibodies; anti-dsDNA, antidouble-stranded DNA; anti-Sm, anti-Smith; anti-RNP, anti-ribonucleoprotein; anti-PCNA, anti-proliferating cell nuclear antigen; IFN interferon; IgG, immunoglobulin G; IgM, immunoglobulin M; IL, interleukin; IP-10, interferon-gamma inducible protein 10; TGF-β, transforming growth factor β; BLyS, B-lymphocyte stimulator; SCF, stem cell factor; SLE, systemic lupus erythematosus.

Signs of autoimmunity in patients with pre-SLE, as described in previous studies.

These signs of autoimmunity can potentially be used to distinguish patients who will eventually be diagnosed with SLE, the patients with pre-SLE, from patients with similar signs and symptoms, but who will never develop SLE. Numerous studies have therefore focussed on different (immunological) biomarkers to aid in the diagnostic process for patients with SLE. Ideally, these studies would use cohorts of patients with pre-SLE to investigate biomarkers that are both accurate and present in prediagnostic phases of SLE. However, as pre-SLE cohorts are very limited, nearly all studies compare patients with an established diagnosis of SLE, fitting the 2019 EULAR/ACR or older classification criteria, with control groups. It is often assumed that the results from these studies are translatable to the pre-SLE phase as well, and therefore have potential relevance for the diagnostic process.

In this review, we give an overview of recent studies investigating biomarkers in patients with an established clinical diagnosis of SLE and their potential for use as diagnostic tools. The use of biomarkers associated with specific organ involvement (ie, skin or renal involvement) is beyond the scope of this review.

## METHODS

We performed a PubMed search with the following search terms: "lupus erythematosus, systemic"[MeSH Terms] AND ("biomarkers"[MeSH Terms] OR "antibodies"[MeSH Terms] OR "immunoglobulins"[MeSH Terms] OR "antibodies"[MeSH Terms] OR "chemokines"[MeSH Terms] OR "cytokines"[MeSH Terms] OR "polymorphism, single nucleotide"[MeSH Terms]) AND ("systemic lupus erythematosus"[Title/Abstract] AND ("biomarker"[Title/Abstract] OR "biomarkers"[Title/Abstract]) AND ("diagnosis"[Title/Abstract] OR "diagnostic"[Title/Abstract])).

Searches were restricted to include articles published from January 2021 up to July 2024.

### Inclusion criteria

Studies that investigated the discriminating potential of 1 or multiple biomarkers in patients with SLE compared with healthy controls (HCs) were included. Only studies that focused on adults, with full-text availability and written in English were included. Relevant studies cited within these articles were also included.

### Exclusion criteria

Studies focussing primarily on the diagnosis of specific organ involvement, such as lupus nephritis or cutaneous lupus erythematosus (CLE), rather than diagnosis of SLE in general, were excluded, as this was beyond the scope of this review.

## RESULTS

The search resulted in 201 articles. After application of the inclusion and exclusion criteria, 61 articles were included. Of 61, 58 included studies note that the patients included in their studies were patients with an established diagnosis of SLE, fitting classification criteria (most commonly the 1997 ACR criteria), or were using public datasets comprising data from patients with SLE fulfilling these classification criteria. Three studies do not explicitly mention which metrics they used to define patients with SLE in their study.

As the pathophysiology of SLE is multifactorial and very complex, we have divided the studies into 5 categories: autoantibodies, other serum markers, markers in other bodily material, circulating RNAs, and gene expression. A simplified overview of these markers and their interconnections is displayed in the Figure.

**Figure:**
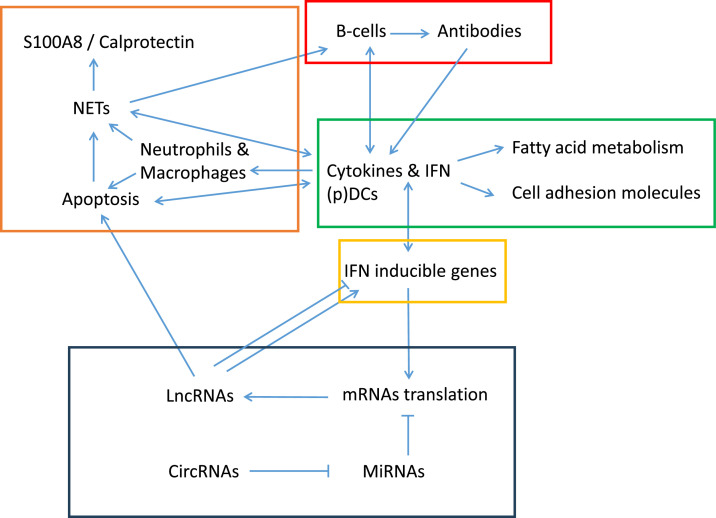
A simplified overview of the different areas of the pathophysiology of SLE and their interconnections, investigated by the articles mentioned in this study. circRNAs, circular RNAs; IFN interferon; lncRNAs, long noncoding RNAs; miRNAs, micro-RNAs; mRNA, messenger RNA; NET, neutrophil extracellular trap; SLE, systemic lupus erythematosus.

### Serum markers

#### Autoantibodies

Over the past 80 years, over 100 different autoantibodies associated with SLE have been identified [20]. In the last couple of years, however, research into the diagnostic potential of autoantibodies seems to have slowed down. We found 6 recent studies investigating the diagnostic use of (auto)antibodies in our search [[21], [22], [23], [24], [25]].

With the use of phage immunoprecipitation sequencing, 1 study used high-throughput DNA sequencing to describe antibodies targeting a peptide from a random peptide library chip (SLE_p27, seq38567 GLYHSNASFRVP). These antibodies were highly distinctive (AUC 0.938) for patients with SLE compared with HCs. It is noteworthy that SLE_p27 outperformed both anti-dsDNA and anti-Sm (AUC of 0.840 and 0.575, respectively) [21] Another study described the presence of anti-neutrophil extracellular trap (NET) antibodies (ANETAs) in patients with SLE, which had moderate diagnostic potential [22]. Interestingly, these ANETAs did not inhibit the immunostimulatory effects of NETs, but rather protected them from degradation, as reported previously [26]. Although they did not find the exact targets of these ANETAs, they noticed that ANETA showed no more than a weak correlation with other tested autoantibodies, such as anti-dsDNA.

Other than focussing on the discovery of new autoantibodies in patients with SLE, some recent studies have focussed on identifying the exact epitopes that previously known autoantibodies react with. A small number of positive ANA tests in patients with SLE, in particular those with an AC-25 or AC-26 pattern, can be explained by the presence of anti-nuclear mitotic apparatus (anti-NuMa) antibodies. Approximately 15% of patients with SLE tested positive for anti-NuMa antibodies and for half of these patients, these were the only autoantibodies that were detected, suggesting potential additional diagnostic value in patients who test negative for conventional autoantibodies [23]. Antimitochondrial antibodies (AMAs) targeting complement C1q binding protein (C1qBP) or mitofusin 1 (MFN-1) are more prevalent in patients with SLE compared with HCs, whereas levels of anti-C1qBP and anti-MFN-1 were not elevated in patients with other diseases associated with the presence of AMAs [24]. Compared with HCs, patients with SLE have increased levels of antibodies targeting the Epstein-Barr virus (EBV) envelope glycoprotein gp350, which might serve as a potential diagnostic biomarker (area under the curve (AUC) 0.804). In addition, the study shows that these antibodies do not appear to be cross-reacting with autoantigens, strengthening the hypothesis that gp350 is a prospective EBV vaccine candidate [25].

One study investigated whether the ratio of immunoglobulin G (IgG) to immunoglobulin M (IgM) ratio would be predictive of progression from incomplete SLE (iSLE) to SLE. They found that patients with iSLE had higher IgG/IgM ratios than HCs, although these ratios were not predictive of progression to SLE in patients with iSLE [27].

Despite the numerous SLE-associated antibodies, the practical use of many of these antibodies is limited, as historically only a small number are deemed to have sufficient added diagnostic value to be incorporated into regular clinical practice [1,28]. Potentially also due to technical improvements leading to the increased accessibility of newer diagnostic methods such as the -omics, more recent research seems to mainly focus on the potential of other diagnostic methodologies [29,30].

### Other serum markers

#### Cytokines and serum proteins

We found 11 studies that have focussed on other serum markers, such as cytokines and other soluble serum proteins [[31], [32], [33], [34], [35], [36], [37], [38], [39], [40]]. Among the cytokines that were found to be upregulated were cytokines that are involved in the induction of inflammation response (interleukin 6 [IL-6], tumor necrosis factor alpha (TNF-alpha), C-C motif chemokine ligand 4 (CCL4) [or macrophage inflammatory protein-1β (MIP-1β)]), and B-cell activation [31,32]. Interestingly, increased serum levels of IL-1 receptor antagonist (IL1RA), which is an anti-inflammatory cytokine, were found as well, with an AUC of 0.938, compared with 0.973 for anti-dsDNA. IL1RA functions by acting as an antagonist to the IL-1 receptor, inhibiting activation of the IL-1 receptor by the proinflammatory IL-1 [31,41]. Serum IL1RA might be elevated in response to the excessive inflammation seen in patients with SLE, as a compensatory modulating agent.

Other studied serum proteins included complement levels, calprotectin, a soluble protein abundantly present in the cytosol of neutrophils (and released in NETs as a major antifungal component), protein hormones (leptin and adiponectin) and several (trans)membrane proteins, in particular involved in cell adhesion (Vascular cell adhesion protein 1 (VCAM-1), E-selectin, L-selectin, sialoadhesin (SIGLEC1)) or vesicular transport (Cav-3, Cav-1) [19,31,34,36,37,39,40]. One study shows an association between persistent low levels of complement proteins C3 and C4 to be associated with progression from iSLE to SLE [40]. VCAM-1 and E-selectin are membrane proteins expressed on endothelial cells, whereas L-selectins are expressed on leukocytes and SIGLEC-1 is expressed on monocytes. These adhesion molecules have been shown to be involved in leukocyte rolling, and their expression is likely increased as a result of inflammation causing endothelial damage [42,43]. Out of these biomarkers, SIGLEC1 had the highest AUC, with an AUC of 0.95, comparable to that of anti-dsDNA (AUC 0.90) [39].

#### Metabolomics

Metabolomics is a relatively new addition to the diagnostic field. Metabolomics is used to study the metabolite composition by measuring numerous metabolites simultaneously [44]. These metabolites can be influenced by diet or microbiota, but can also be changed as a result of inflammation [45,46].

Six recent studies investigated changes in metabolomics in patients with SLE compared with HCs [[47], [48], [49], [50], [51], [52]]. In general, lipid and fatty acid metabolism seem most affected in patients with SLE in these studies. Multiple studies suggest that changes in the lipid and fatty acid metabolism might result in the increased prevalence of cardiovascular disease in patients with SLE, which is a major cause of mortality in patients with SLE. These 6 studies found multiple metabolites, either single metabolites or multiple metabolites combined into a multivariate model, with an AUC of 0.950 or larger, for patients with SLE compared with HCs, which suggests great diagnostic potential.

#### Proteomics

Whereas metabolomics studies the composition of metabolites in patients, proteomics focusses on the proteome, the collection of proteins in these patients. Three recent studies investigated the proteome of patients with SLE and found that a number of proteins were significantly changed in patients with SLE [47,53,54]. These proteins, such as Interferon-induced protein with tetratricopeptide repeats 3 (IFIT3), Tumor necrosis factor receptor II (TNF-RII), and Amyloid A1, mainly reflected organ damage and inflammation, with multiple interferon-induced proteins being found differentially expressed between patients with SLE and HCs.

Some of the studies mentioned above show correlations of the investigated biomarkers with conventional diagnostic markers, such as anti-dsDNA and complement factors C3 and C4. Three studies also compared the AUC of the investigated biomarker to that of anti-dsDNA and complement factors [37,39,41]. In all three cases, the AUC of the investigated biomarkers was similar to that of anti-dsDNA and outperformed the AUC of complement factors.

### Markers in other bodily materials

Although most diagnostics involve tests that are performed using a blood or serum sample, 5 studies have also investigated the use of other bodily materials as a diagnostic of SLE [[55], [56], [57], [58], [59]]. Three studies have investigated urine samples, whereas the diagnostic potential of the composition of pleural effusion and the microbiome was investigated by one study, respectively.

Similar to blood samples, urine samples are easy to collect and are commonly used in current clinical practice. The main use of urine diagnostics in current clinical practice is to screen for lupus nephritis. For the purpose of this review, we only included studies investigating the use of diagnostics on urine samples for the diagnosis of SLE in general and excluded studies investigating the use of urine samples to diagnose lupus nephritis in patients with established SLE.

The first study found that levels of several proinflammatory proteins were elevated in the urine of patients with SLE, compared with HCs [55]. The second study showed that patients with SLE also have increased urinary levels of beta-2 glycoprotein 1, which is also known as one of the targets of antiphospholipid antibodies. Interestingly, the authors found an AUC of 0.946 for patients with SLE compared with HCs, with similar levels of urinary beta-2 glycoprotein 1 in patients with or without lupus nephritis [56]. The third study showed increased levels of S100A8 (S100 calcium-binding protein A8) in urine, as well as in serum and saliva of patients with SLE [57]. Together with S100A9, S100A8 forms the heterodimer calprotectin, which itself also has been shown to be elevated in the serum of patients with SLE. Similar to calprotectin, elevated serum levels of S100A8 are thought to be associated with NETosis, as it is abundantly present in the cytosol of neutrophils [60].

A study investigating levels of complement C3 and C4 in pleural effusion found that C3 and C4 could be used to distinguish pleural effusion caused by SLE from pleural effusion of other causes, with lower levels of C3 and C4 being found in the pleural effusion of patients with SLE [58]. Although these levels of C3 and C4 were significantly lower than in pleural effusion caused by malignancy or infection, they were not significantly different from levels of C3 and C4 seen in the pleural effusion caused by fluid overload.

One study investigated the composition of the microbiome of patients with SLE using 16S rRNA analysis [59]. Using a machine learning model, they were able to create a predictive model with an AUC of 0.930. Presence of *Escherichia fergusonii*, a pathogenic bacterium known to cause urinary tract infections, was found as the most important contributing factor to the model [61].

### Circulating RNAs

Several studies have investigated the diagnostic properties of circulating RNAs. These can broadly be divided into 3 different categories: micro-RNAs (miRNAs), circular RNAs (circRNAs), and long noncoding RNAs (lncRNAs).

#### Micro-RNAs

MiRNAs are short noncoding RNA fragments that regulate protein expression by blocking the translation of the corresponding messenger RNA (mRNA) by binding to it. Depending on the function of the protein coded by the corresponding mRNA, miRNAs can have both immunostimulating and immunosuppressive effects [62].

One study found increased levels of miR-21 and miR-146a in patients with SLE, compared with HCs [63]. They note that miR-21 is associated with T-cell activation and miR-146a is associated with type 1 interferon (IFN) response. A different study found increased levels of miR-181a and decreased levels of miR-223 in patients with SLE, compared with HCs [64]. MiR-181a plays a role in lymphocyte differentiation, and miR-223 influences granulocyte differentiation [65,66]. Another study showed increased levels of miR-124-3p and miR-377-3p in patients with SLE [67]. The authors suggest that both these miRNAs might inhibit the transcription of *EGR1*, which functions as a transcription regulator and has been shown to be downregulated in patients with SLE, although they did not experimentally confirm this [68]. A. found miR-183-5p to be upregulated in patients with SLE. MiR-183-5p targets Foxo1, which showed inverse correlation with miR-183-5p expression [69]. It has been suggested that Foxo1 downregulation is one of the important causes of Th17 dysregulation [70].

Despite the significant alterations in levels of miRNAs found by these studies, in general, they appear not to be very specific for SLE, as many of these have also been associated with different types of cancer and other diseases [71]. Therefore, these alterations might not represent changes specific to SLE, but rather a change from the normal homeostasis, as seen in a multitude of diseases. For example, as a review by Jenike and Halushka [71] points out, miR-21 is one of the most abundant miRNAs and has been claimed as a biomarker in at least 29 different diseases, and therefore cannot be considered a useful biomarker.

#### Circular RNAs

Whereas miRNAs are suggested to inhibit mRNA transcription, circRNAs act as “sponges” for miRNAs, therefore limiting the modulating effect of miRNAs [72]. One study found that circPTPN22 is downregulated in patients with SLE, as well as in patients with rheumatoid arthritis [73]. A follow-up study by the same research group found that circPTPN22 acts as a sponge for miR-4689, which targets S1PR1, an important regulator of immune cell migration, differentiation, and proliferation [74].

One study found increased expression levels of 3 circRNAs (hsa_circ_0082688, hsa_circ_0082689, and hsa_circ_0008675). A model using a combination of 2 of these circRNAs resulted in an AUC of 0.740 for distinguishing patients with SLE and HCs [75]. In another study by the same group, they did not reproduce these findings, but instead describe lowered levels of 4 different circRNAs (hsa_circ_0000175, hsa_circ_0044235, hsa_circ_0068367, and hsa_circ_0001947) [76]. In addition, a different study found that hsa_circ_0006689 was overexpressed in patients with SLE compared with HCs with an AUC of 0.713, and another study found overexpression of circETS1 in patients with SLE, with an AUC of 0.873 [77,78]. The lack of reproducibility and unclear pathophysiological mechanisms of these circRNAs limit their diagnostic potential.

#### Long noncoding RNAs

LncRNAs are commonly defined as RNAs with a length of over 200 nucleotides that are not translated into functional proteins. Many lncRNAs are considered transcriptional noise, although increasing numbers of lncRNAs have been shown to have biological functions, mainly with a regulatory or architectural role in cell biology [79].

We found 7 studies investigating the use of lncRNAs for the diagnosis of SLE [[80], [81], [82], [83], [84], [85], [86]]. The function of the lncRNAs mentioned in these studies is often unclear, but the lncRNAs reported in these studies are commonly suggested to have an inflammation regulatory function. Two studies report lncRNAs associated with apoptosis pathways [83,86]. One study found increased expression levels of lncRNA SNHG1, which is found to negatively regulate tumour suppression genes such as p53, and found that lncRNA SNHG1 overexpression was associated with an increased apoptosis rate in peripheral blood mononuclear cells (PBMCs)s in patients with SLE. Another study found an increased expression of lncRNA H19, which they presume to target miRNA 19-b (miR-19b). miR-19b has been shown to enhance the proliferation of PBMCs and induce apoptosis resistance, whereas lncRNA H19 can inhibit the function of miR-19b.

### Gene expression

Several recent studies have focussed on gene expression profiles in SLE [[87], [88], [89], [90], [91], [92], [93], [94], [95], [96], [97], [98]]. Many of these studies used similar methodologies, using existing datasets from previous trials, that have been published in an online repository with the Gene Expression Omnibus (GEO) database. These new studies use the gene expression data series (GSE) from these trials to identify interesting differentially expressed genes (DEGs), validate the DEGs, often using other GSEs, and then validate the findings using RT-qPCR in a (small) local cohort. Following this, Kyoto Encyclopedia of Genes and Genomes and Gene Ontology analyses are often used to further investigate the function of the genes found by these analyses.

Many of the DEGs reported by these studies are known interferon-inducible genes, such as *IFI44, IFI44L, MX2, IFI27*, and *EPSTI1* [[88], [89], [90],[92], [93], [94], [95], [96], [97]]. Some of these genes are also included in commonly used combinations to calculate the so-called ‘interferon signature’ [99]. One study used Mendelian Randomisation to predict cytokine levels using publicly available genome-wide association study (GWAS) data. Their main finding suggests an increase in levels of IL-4 in patients with SLE, identifying it as a potential biomarker, although they mention this has to be further investigated in experimental studies [100].

It is noteworthy to highlight a study by Khunsriraksakul et al [98]. In this study, they performed a multiancestry and multitrait meta-analysis of 12 SLE GWAS studies, which included over 20,000 patients with SLE and almost 700,000 HCs. They found 16 new loci associated with SLE, and integrated the differentially expressed genes into a polygenic risk score (PRS) to evaluate the diagnostic potential. Nevertheless, the PRS on its own had an AUC of 0.65 to 0.69, whereas the more conventional combination of ANA and anti-dsDNA had an AUC of 0.72-0.73. Combining both only increased the AUC slightly to 0.75.

#### Methylation levels

Methylation of gene promotors is an important epigenetic factor in gene expression regulation, that can both increase or inhibit the expression of the associated gene, based on the binding site on the DNA [101]. Two studies investigated the methylation levels of specific genes in PBMCs of patients with SLE, compared with HCs [102,103]. Both of these studies found increased methylation of tumour suppressor genes in PBMCs, potentially indicating decreased function of these genes. One of these studies showed increased methylation of the *RUNX3* gene, a known tumour-suppressor gene that is frequently deleted or silenced in different types of cancers [102,104,105]. The other study found increased methylation levels of *CDKN2A* promotors, which encodes 2 tumour suppressor genes, p14 and p16, and has been shown to modulate production of inflammatory cytokines [103].

## DISCUSSION

There is a need for additional tools to expedite the diagnostic process for patients with SLE, as the current diagnostic process often leads to diagnostic and treatment delays [2,3]. In this review, we evaluated recent developments in the use of biomarkers for the purpose of diagnostics in patients with SLE.

Antibodies are one of the few immunological manifestations that have been shown to actually be present before the diagnosis of SLE is eventually made, as Arbuckle et al [5] showed previously in a longitudinal cohort study. Combined with the ease of investigating the presence of autoantibodies, they have therefore historically been a prime target for the discovery of new diagnostic biomarkers. However, over half of the studies investigating autoantibodies included in this review mainly focussed on elucidating the exact antigens of known autoantibodies, rather than the presence of undiscovered autoantibodies. Nevertheless, the number of SLE-associated antibodies has grown significantly since the study by Arbuckle et al [28]. It would be interesting to repeat a study similar to that of Arbuckle et al, which includes multiple SLE-related autoantibodies that were not included in the original study to investigate the sensitivity of other autoantibodies or combinations of autoantibodies in the preclinical phases of SLE.

An aberrant process of apoptosis and increased prevalence of NETs are known phenomena in patients with SLE. Previous studies have shown an increased prevalence of NETosis-prone low-density granulocytes in patients with SLE [106]. Interestingly, increased levels of calprotectin, a soluble protein abundantly present in the cytosol of neutrophils and an important component of NETs, are found in the serum of patients with SLE [34]. Increased levels of S100A8, one of the 2 major components of calprotectin, are also found in the serum, as well as the urine and saliva of patients with SLE [57,60]. S100A8 might serve as an indirect marker of NETosis activity, which might be useful in the diagnostic process or can potentially be used to identify different subsets of patients with SLE for treatment selection.

Cytokines play an important part in the regulation of the autoimmune pathophysiology. Production of cytokines is influenced by many factors and can also influence different parts of the immune system. The studies reviewed in this article show increased prevalence of multiple proinflammatory cytokines as well as altered levels of cell adhesion molecules such as selectins, potentially facilitating extravasation of leukocytes into tissues [[31], [32], [33], [34], [35], [36], [37], [38]]. In addition, changes in fatty acid metabolism were found in patients with SLE using metabolomics analysis. There are some caveats that should be taken into consideration while interpreting the metabolomics data. First, all 6 of these included studies were conducted in China. Because of globally different dietary habits and the influence that diet can have on the metabolic profile, combined with differences in genetic background, this might limit the global applicability of these models [107]. Second, metabolism can be altered by disease-modifying drugs, including glucocorticoids such as prednisone, which are commonly given to patients with SLE [108]. Although these recent studies do report the use of medication, the use of different types of medication might influence their results.

It would be interesting to investigate whether these changes in metabolism, as well as the potentially related increased cardiovascular risk observed in patients with SLE can be observed during pre-SLE as well [109]. Overall, cytokines and metabolomic analyses result in promising AUCs when comparing patients with SLE to HCs. If future studies can confirm these findings in pre-SLE, this would confirm their potential as a diagnostic tool for SLE.

Levels of multiple different microRNAs, their presumed inhibitors circRNAs, and lncRNAs were found to be altered in patients with SLE in comparison to HCs. Nevertheless, their overall specificity is lacking, as multiple of these circulating RNAs have been associated with a multitude of different diseases. For example, miR-21 has been associated with over 20 different diseases [71]. We hypothesise that changes in these circulating RNAs are mainly a reflection of nondisease-specific changes in homeostasis, rather than a reflection of disease-specific changes. This would likely limit their use for diagnostics in SLE, as it might show signs of nonspecific disturbance of regular homeostasis, but would not aid the diagnostic process significantly.

A number of genes associated with an increased risk of development of SLE have been found in previous studies, although this only fully explains the presence of SLE in a small number of familial cases [110]. In particular, the increased expression of IFN-inducible genes, commonly referred to as the IFN-signature, has been well-established in (a subgroup of) patients with SLE, as well as some other autoimmune diseases like Sjogren’s syndrome [111,112]. Most of the studies investigating differential gene expression included in this review also found increased expression levels of IFN-related genes, such as *IFI44*. Nevertheless, the diagnostic accuracy of the IFN-signature has so far been deemed to be insufficient to have been incorporated in the standard diagnostic process for SLE.

One study using a large multiethnic cohort managed to only marginally increase the diagnostic performance of conventional diagnostics using a PRS [98]. Potential reasons for this relative failure are differences between different ethnic backgrounds, resulting in too much heterogeneity in the cohort, or the prevalence of different pathophysiological profiles resulting in different gene expression profiles. Similar to the other areas studied, it would be interesting to investigate changes in genetic expression profiles in pre-patients with SLE, to answer the question whether these changes are drivers of pathology, accumulating over time to eventually cause disease, or merely an epiphenomenon resulting from other pathophysiological manifestations, such as increased interferon production. Although the diagnostic performance of gene expression profiles might be limited, identification of these dysregulated pathways provides new potential drug targets [113]. Furthermore, identification of which pathways are dysregulated in individual patients has potential for use in precision medicine, tailored to the immunological changes in the individual.

It is noteworthy that many of the studies investigating gene expression profiles included in this review used datasets from previous cohorts, accessible via the GEO database [[87], [88], [89], [90], [91], [92], [93], [94], [95], [96], [97]]. Most of these studies do not mention how or why they selected the specific GSE they chose to use for their analysis. Future studies would benefit from describing this selection process more clearly to reduce the suggestion of a selection bias. In addition, future studies should report more clearly what type of cells are used in the GSEs they used for their study. GSEs in the GEO database are most often derived from PBMCs or whole blood, with some smaller datasets investigating DEGs in other cell lines, such as CD4+ T-cells or B-cells. Some studies included in this review are using data from paediatric SLE cohorts without stating so [89,92,96]. In one study, it seems that first-degree relatives of patients with SLE, included in the cohort GSE24706, are classified as patients with SLE [97]. Comparing results from different cell lines and different populations might influence results and should be clearly stated if done so.

In our manuscript, we tried to accurately display the findings that were relevant to our research question in all included studies. This meant that for all included studies, we primarily highlighted the findings that were related to diagnostic potential for patients with SLE. We emphasise that for some of the included studies, showing diagnostic potential for patients with SLE was not the main goal of their study. This has resulted in the omission of important or main findings of some of the studies in our review, as they did not fit within our narrow research question. In addition, we did not include articles discussing biomarkers primarily aimed at predicting specific organ involvement, such as lupus nephritis or CLE, as this was beyond the scope of our review. Although our review focusses on biomarkers predicting the development of SLE, it is noteworthy that approximately 15% of patients with CLE progress to SLE [114]. A review by Zhu et al [115] describes an association between the presence of ANAs, anti-dsDNA, anti-Sm, anti-RNP autoantibodies and an elevated erythrocyte sedimentation rate with progression from CLE to SLE. In our search, we found no additional studies describing biomarkers that predict the progression from CLE to SLE.

Overall, with our review, we have not found any recent studies into biomarkers that are likely to change the diagnostic process in the short term. Nevertheless, some studies show promising results, meriting further investigation. For example, the peptide SLE_p27 outperformed anti-dsDNA and anti-Sm in diagnostic accuracy [21]. Similarly, the serum levels of SIGLEC1 showed a good sensitivity and specificity for SLE, although the authors note that SIGLEC1 is a surrogate marker for IFN-I pathway activation and elevated levels are also found in other inflammatory diseases or infections [39]. In addition, multiple studies investigating metabolomics in patients with SLE found AUCs of 0.95 or higher for several biomarkers, although these different studies do not identify the same metabolomic biomarkers [[47], [48], [49], [50], [51], [52]].

Most of the studies included in this review show differences between HCs and patients with an established diagnosis of SLE, often already undergoing treatment which might also influence the results. It often is assumed that the findings of the studies comparing biomarkers in patients with SLE with HCs are transferrable to the early diagnostic phases, although this is rarely investigated properly. Studies investigating immunological changes in patients in the period leading up to their SLE diagnosis are very limited [[5], [6], [7], [8], [9], [10], [11], [12], [13], [14], [15], [16], [17], [18], [19]]. A small number of these studies have specifically investigated predictors of progression from incomplete SLE to definite SLE, fulfilling classification criteria [[14], [15], [16], [17], [18], [19]]. A review by Lambers et al [116] provides a more extensive overview of these studies. These studies have identified some potentially predictive biomarkers for progression in this group, such as increased levels of anti-dsDNA and lowered C3 levels. However, these studies represent a very selective population and commonly use small cohorts with a limited number of patients with pre-SLE.

One major improvement in diagnostic studies would be to perform studies using (serum) samples from patients with pre-SLE, although this has significant methodological and logistic challenges, requiring large (population-based) cohorts with regular collection of bodily material. The 2023 EULAR SLE recommendations also named the establishment of pre-SLE cohorts as a key part of the future research agenda, to better be able to identify patients at risk to develop SLE and gain further insight into the disease development stage [117]. The creation of such population-based cohorts would likely be a costly endeavour, but could contribute to research projects not only in the field of SLE, but for many different diseases and would therefore serve many purposes. In addition, the increasing potential and accessibility of artificial intelligence models also have the potential to aid in the development of diagnostic multivariate models and the recognition of potential pre-patients with SLE. To our knowledge, no data on newly established pre-SLE cohorts had been reported at the time of our search. It would be very interesting to further investigate promising biomarkers such as SLE_p27 and SIGLEC1 in pre-SLE cohorts. Although more elaborate to measure, changes in metabolomics in patients with pre-SLE could also grant further insight in the pathophysiological process in the early stages of SLE.

## Funding

The authors confirm that they received no funding for this article.

## CRediT authorship contribution statement

**Tammo Brunekreef:** Writing – review & editing, Writing – original draft, Visualization, Investigation, Conceptualization. **Henny Otten:** Supervision, Conceptualization. **Jacob M. van Laar:** Supervision, Conceptualization. **Maarten Limper:** Supervision, Conceptualization.

## Competing interests

The authors declare that they have no known competing financial interests or personal relationships that could have appeared to influence the work reported in this paper.
